# When Stones Escape: A Rare Radiologic Finding of Retroperitoneal Stone Extrusion Following American Association for the Surgery of Trauma (AAST) Grade IV Blunt Renal Trauma in a Chronically Hydronephrotic Kidney

**DOI:** 10.7759/cureus.96501

**Published:** 2025-11-10

**Authors:** Youssef Berday, Fatimazohra El Yaagoubi, Manal El Moujahid, Imane Karchali, Lina Amzazi, Samia Abbar, Zakaria Chahbi, Soukaina Wakrim

**Affiliations:** 1 Radiology, Souss-Massa University Hospital Center, Agadir, MAR; 2 Radiology, Hospices Civils de Lyon, Lyon, FRA

**Keywords:** computed tomography, double-j stent, hydronephrosis, renal trauma, retroperitoneal space, stone extrusion, urinoma, urolithiasis

## Abstract

Traumatic injury of a hydronephrotic kidney is uncommon, and extrusion of calculi beyond the renal capsule is exceptionally rare. We describe a middle-aged man with severe left hydronephrosis who sustained a low-impact fall, resulting in an American Association for the Surgery of Trauma (AAST) grade IV renal laceration. Contrast-enhanced CT revealed urinary extravasation, inflammatory urothelial enhancement consistent with uretero-pyelitis, and multiple displaced calculi within the retroperitoneum. A double-J stent was inserted after the initial scan. Follow-up CT demonstrated a well-circumscribed perirenal collection with peripheral enhancement during the venous phase, consistent with a superinfected urinoma. Conservative management led to full recovery. This case highlights how chronic obstruction weakens the renal parenchyma, predisposing to rupture and stone migration even after minor trauma.

## Introduction

Renal trauma accounts for 5-10% of all abdominal injuries, with blunt mechanisms being predominant [1]. Most occur in otherwise healthy kidneys; however, kidneys affected by pre-existing abnormalities (such as hydronephrosis, urolithiasis, or other uropathies) appear more vulnerable to injury, even after relatively minor trauma [2].

In hydronephrotic kidneys, chronic obstruction leads to progressive cortical thinning and elevated intrapelvic pressure, resulting in a weakened renal parenchyma that becomes highly susceptible to rupture even after minor increases in pressure or trauma [3]. To the best of our knowledge, traumatic extrusion of renal calculi through a cortical rupture has not been previously reported, making this an exceptional and educational radiologic case. We report a case of American Association for the Surgery of Trauma (AAST) grade IV [4] blunt renal trauma leading to stone migration beyond the capsule, emphasizing its characteristic computed tomography (CT) features, pathophysiological mechanisms, and management strategy.

## Case presentation

A 35-year-old man with a history of intermittent left flank pain presented after a fall from standing height onto his left side. He was hemodynamically stable but complained of severe flank pain and gross hematuria. Laboratory tests revealed mild anemia and a slight rise in serum creatinine. The patient reported no known history of urinary tract disease and had never undergone prior imaging evaluation. Hydronephrosis was therefore an incidental radiologic finding at the time of trauma assessment, with no documented antecedent diagnosis or follow-up.

Imaging findings

**Figure 1:**
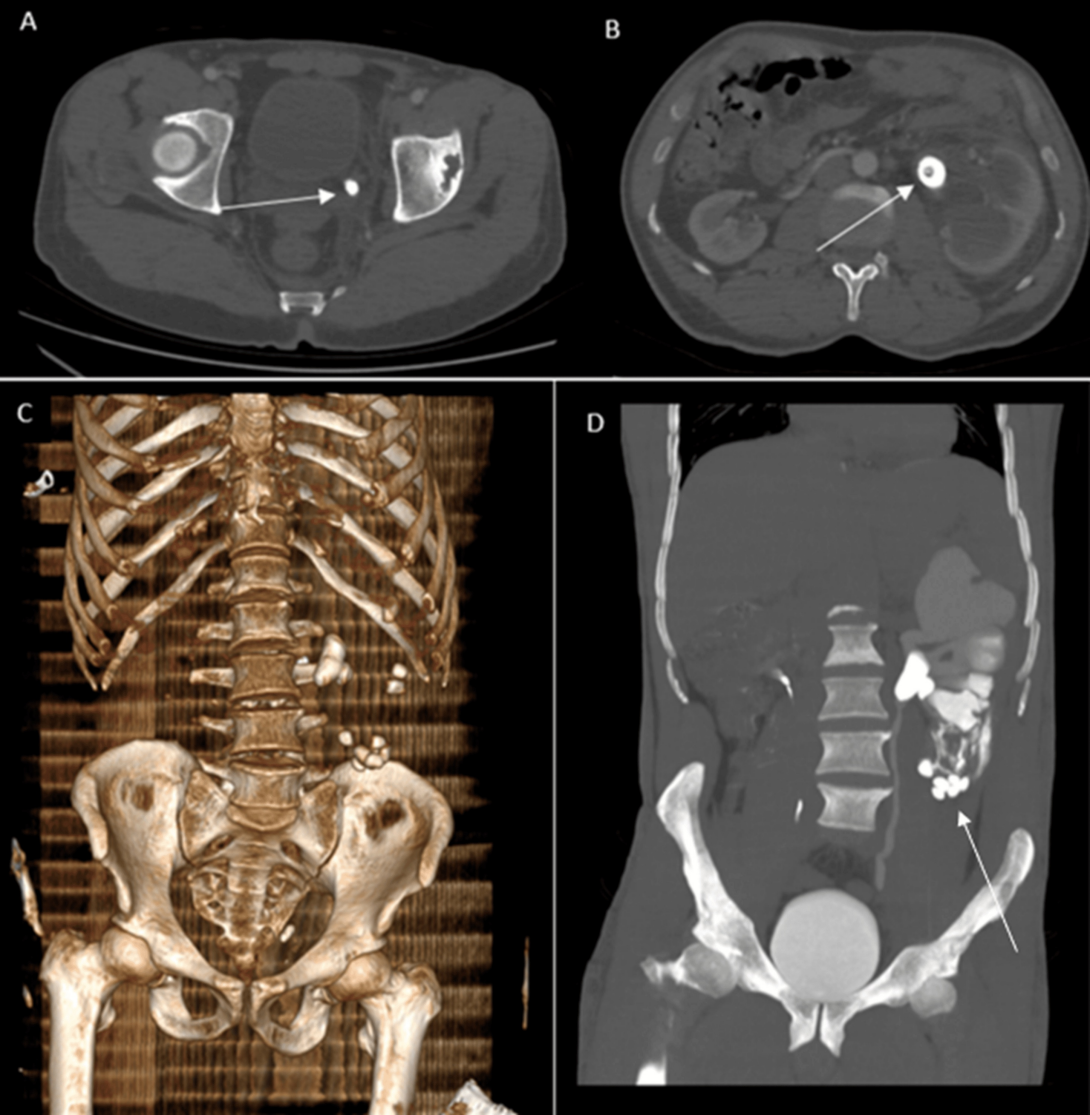
Imaging studies (A) Axial CT image in bone window during the arterial phase showing a distal ureteral stone; (B) Axial CT image in bone window during the arterial phase, demonstrating a large staghorn calculus within the renal pelvis; (C) Three-dimensional volume-rendered reconstruction depicting multiple renal and ureteral calculi; (D) Coronal maximum intensity projection (MIP) reconstruction in the delayed phase showing multiple calculi and marked left hydronephrosis. Some stones appear extrarenal, displaced beyond the renal capsule.

Contrast-enhanced CT revealed a markedly enlarged left kidney (17 × 8 × 6 cm) with severe calyceal dilatation and cortical thinning. The degree of dilatation corresponded to grade IV hydronephrosis, characterized by marked ballooning of the pelvicalyceal system with severe cortical thinning, consistent with long-standing obstruction. Two obstructive calculi were seen at the pelvi-ureteric junction and distal ureter, the largest measuring 4 cm (~1100 HU) and coralliform in shape (Figure 1).

**Figure 2:**
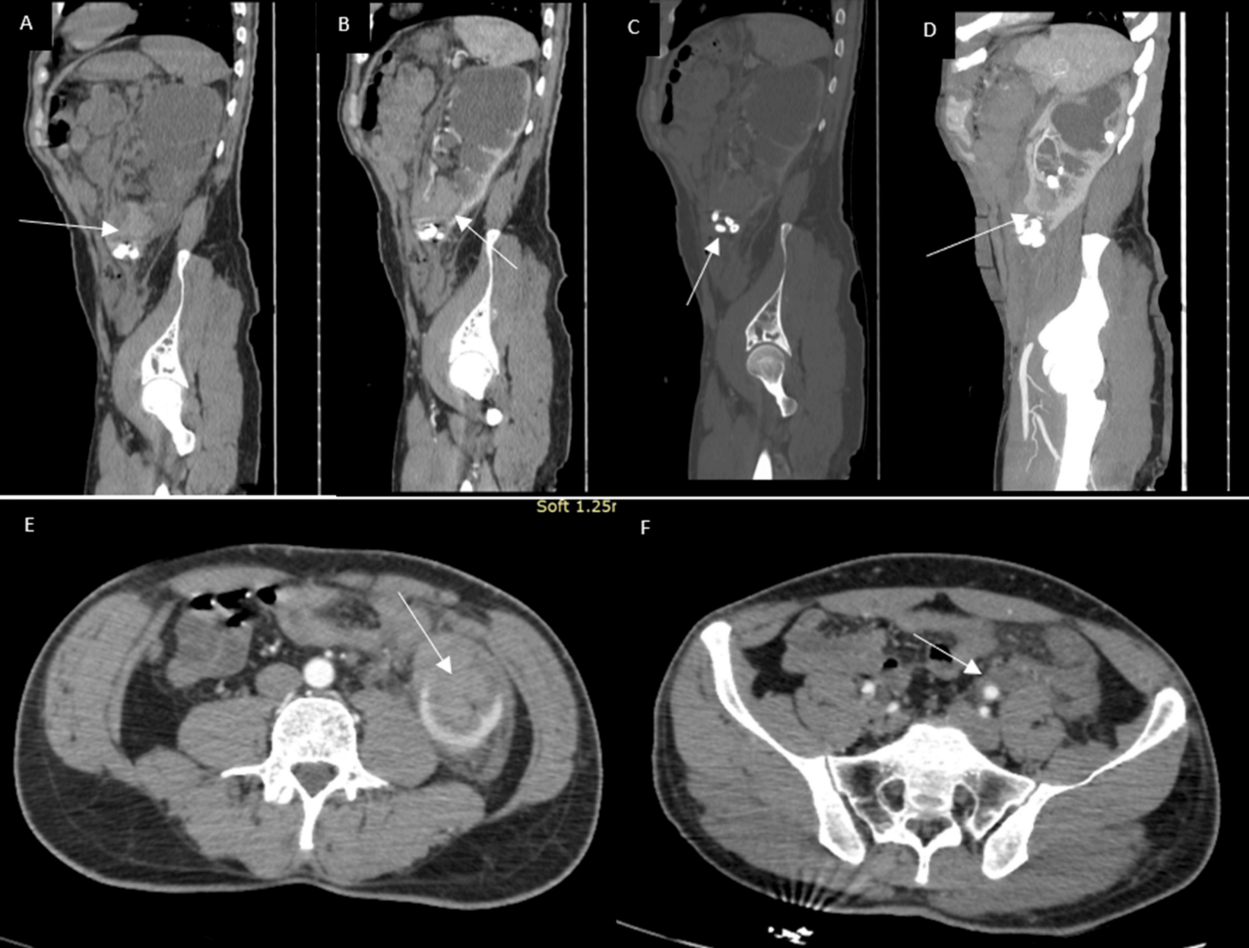
CT images (A) Sagittal reformatted non-enhanced CT image showing a spontaneously hyperdense hematoma within the lower calyceal group; (B) Sagittal CT reformatted image in the arterial phase, showing a hematoma involving the lower calyceal group, passing through a cortical defect at the lower pole; (C) Sagittal CT reformatted image in the arterial phase, bone window, demonstrating extrarenal calculi; (D) Sagittal maximum intensity projection (MIP) CT reformatted image in the arterial phase showing a cortical discontinuity at the lower pole, allowing better visualization of the extrarenal position of the calculi; (E) Axial CT image in the arterial phase showing the parenchymal laceration at the lower pole associated with a perirenal hematoma; (F) Axial CT image in the arterial phase, demonstrating a retroperitoneal hematoma extension.

**Figure 3:**
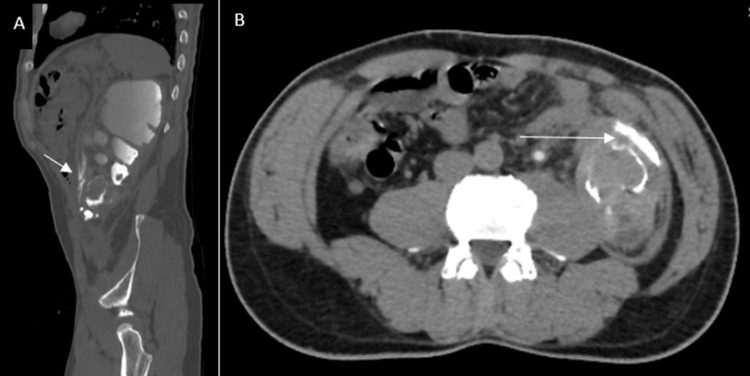
Additional CT images (A) Sagittal CT reformatted image in bone window during the delayed phase showing contrast extravasation tracking around the perirenal hematomas; (B) Axial CT image in the delayed phase, demonstrating contrast medium extravasation adjacent to the lower pole laceration.

There was diffuse urothelial enhancement of the collecting system and proximal ureter, suggesting associated uretero-pyelitis. A 35 mm cortical laceration at the lower pole extended into the collecting system, with urinary extravasation visible on delayed images (Figures 2, 3).

Multiple hyperdense foci corresponding to extruded calculi were identified outside the renal parenchyma, within the perirenal fat and retroperitoneal space, accompanied by a moderate perirenal hematoma and urinoma tracking along the left paracolic gutter (Figures 2, 3). The right kidney was normal except for a few Bosniak I cysts, and no skeletal or visceral injuries were present.

**Figure 4:**
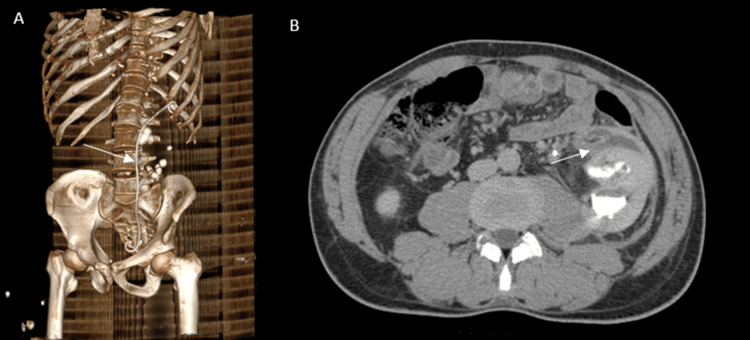
CT images after placement of the double-J stent (A) Three-dimensional volume-rendered reconstruction from the follow-up CT showing a double-J ureteral stent in situ; (B) Axial CT image in the venous phase demonstrating perirenal fluid collections with peripheral enhancement.

The patient was treated conservatively with analgesia, antibiotics, and close monitoring. A double-J ureteral stent (Coloplast, Humlebæk, Denmark) was inserted following the initial CT to relieve obstruction and promote urinary drainage. Follow-up contrast-enhanced CT performed several days later demonstrated collection of the previously diffuse hemo-urinoma into a well-defined perirenal collection with smooth peripheral enhancement during the venous phase, consistent with a superinfected urinoma (Figure 4).

The patient’s condition progressively improved, and he was discharged with preserved renal function. Elective management of residual lithiasis and hydronephrosis was planned subsequently.

## Discussion

The revised AAST renal injury grading system provides a comprehensive framework for classifying renal trauma according to the extent of parenchymal disruption, urinary extravasation, and vascular injury [4]. Grades I to III represent minor to moderate lesions, ranging from contusions or superficial cortical lacerations without urinary leakage to deeper lacerations confined to the parenchyma. Grade IV injuries are characterized by lacerations extending into the collecting system or segmental vascular injuries resulting in contained hematomas. Grade V includes the most severe patterns, such as a shattered kidney or complete avulsion of the renal hilum. This standardized classification allows consistent communication among radiologists, urologists, and trauma surgeons, facilitating therapeutic decision-making and prognostic evaluation.

In the present case, the cortical laceration reached the collecting system, fulfilling the criteria for an AAST grade IV renal injury. In this patient, chronic hydronephrosis and cortical thinning predisposed the kidney to rupture under minimal trauma. In hydronephrosis, sustained elevation of intrapelvic pressure leads to progressive cortical thinning and structural alteration of the renal parenchyma, thereby diminishing its normal compliance and potential resilience to mechanical stress [5]. The impact likely produced a tear at the lower pole, creating a pathway through which intrarenal calculi were expelled into the retroperitoneal space.

Extrusion of renal calculi through a traumatic cortical rupture represents an exceptionally rare event. To the best of our knowledge, no similar case has been previously reported in the literature. A structured literature search was performed across PubMed, Embase, and Google Scholar (1980-2025) using the keywords renal trauma, renal calculi, stone extrusion, and extrarenal migration. This search yielded no prior reports of post-traumatic stone extrusion, underscoring the uniqueness of the present case. The diagnosis relies primarily on CT, which can simultaneously demonstrate parenchymal disruption and urinary extravasation [6]. Delayed-phase images are critical for identifying collecting-system leaks and associated urinoma.

CT remains the gold standard for evaluating renal trauma and determining injury grade according to AAST criteria [7,8]. Non-contrast CT accurately identifies hyperdense hematomas and high-attenuation calculi (>1000 HU), whereas contrast-enhanced multiphasic imaging provides a detailed assessment of parenchymal disruption, vascular injury, and urinary extravasation. The corticomedullary and nephrographic phases outline lacerations and perfusion defects, while the delayed excretory phase confirms collecting-system rupture or urinoma formation. Multiplanar reconstructions and 3D volume-rendered images are especially useful for evaluating the spatial relationship between lacerations, vessels, and stones [9].

Ultrasound (US) serves as a convenient first-line modality, particularly in hemodynamically unstable patients or when CT is unavailable. It detects renal enlargement, hydronephrosis, and perirenal collections suggestive of urinoma or hematoma, but its sensitivity for small cortical lacerations and active bleeding remains limited [10].

Contrast-enhanced ultrasound (CEUS) improves the performance of standard US by enabling real-time evaluation of renal perfusion. It can demonstrate cortical devascularization, pseudoaneurysm, or active contrast extravasation comparable to CT angiography, and is particularly helpful for follow-up without radiation exposure [11].

Although intravenous urography (IVU) has largely been replaced by CT urography, it retains value when CT is contraindicated, providing information on renal excretory function and revealing delayed or absent opacification in cases of collecting-system rupture or devascularization [7].

Magnetic resonance imaging (MRI) offers excellent soft-tissue contrast and is especially suited for pregnant women, pediatric patients, and individuals with iodinated contrast allergy. T1- and T2-weighted sequences characterize hematomas and urinomas, while post-contrast and diffusion-weighted imaging delineate enhancement defects and differentiate viable from necrotic tissue [9].

Finally, interventional radiology plays a vital diagnostic and therapeutic role. Selective angiography and endovascular embolization effectively control bleeding while preserving renal parenchyma, and percutaneous drainage or nephrostomy may be used to treat urinomas, abscesses, or persistent urinary leaks [10].

Together, these complementary modalities form a multimodal imaging and treatment framework that allows precise lesion characterization, risk stratification, and organ-preserving management of renal trauma.

In hemodynamically stable patients, even high-grade (AAST IV) renal injuries can often be managed conservatively [12]. Conservative management typically includes bed rest, antibiotics, and urinary diversion-either by ureteral stenting or percutaneous nephrostomy to control leakage and preserve renal function. Follow-up imaging is essential to confirm resolution of hematoma or urinoma and to guide the timing of definitive stone treatment.

## Conclusions

This case underscores a rare consequence of minor blunt trauma in a chronically obstructed kidney. Although the findings are conclusive, a limitation of this report remains the absence of prior imaging, which likely delayed recognition of the chronic hydronephrosis before trauma. While spontaneous rupture of a hydronephrotic kidney can present with similar findings, it was ruled out given the clear traumatic mechanism and distinct cortical disruption on CT. Awareness of potential stone extrusion through a traumatic laceration is essential for radiologists and urologists. CT imaging plays a pivotal role, as it can simultaneously depict acute injury, chronic obstruction, and extruded calculi. Complementary imaging modalities, such as ultrasound, MRI, and intravenous urography, can refine diagnosis and follow-up, while interventional radiology offers minimally invasive solutions for complications. Prompt diagnosis allows appropriate conservative management and defers definitive treatment of the underlying obstruction once the acute phase resolves.
